# Max Wilson and the Principles and Practice of Screening for Disease

**DOI:** 10.3390/ijns6010015

**Published:** 2020-02-29

**Authors:** Kate Hall

**Affiliations:** UK Newborn Screening Laboratories Network, UK; kate@pdsoft.co.uk

**Keywords:** Wilson and Jungner, principles of screening, population screening

## Abstract

The name Wilson will be forever associated with co-author Jungner and ten principles of population screening published in 1968 by the World Health Organisation (WHO) as Public Health Papers No 34. These principles have since been used, modified or extended throughout much of Europe and beyond. Very little was known about Dr. J.M.G. Wilson and his life and how he came to write this monograph until the Silver Jubilee meeting of the International Society for Neonatal Screening held in The Hague in 2016. The opening session was chosen to be ‘The Wilson and Jungner criteria for screening for disease’.

## 1. Introduction

**Figure 1:**
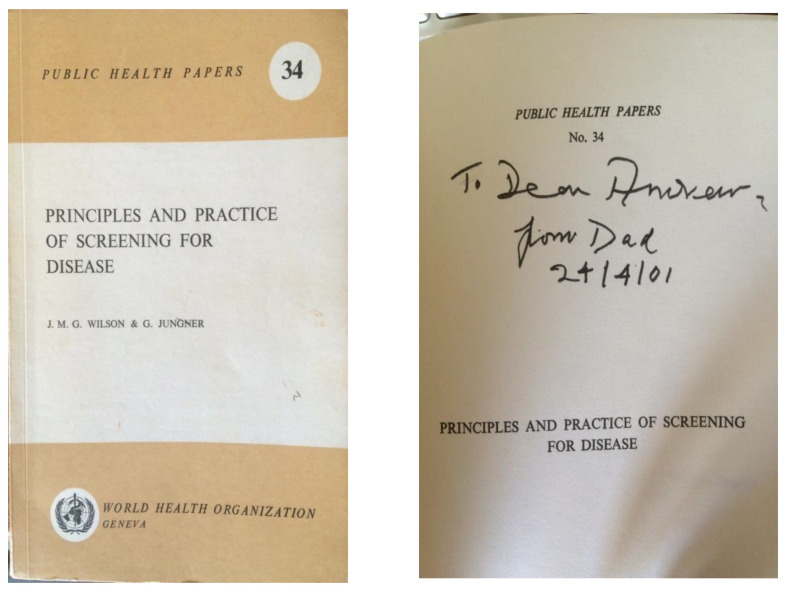
J.M.G. Wilson’s personal copy given to his eldest son Andrew.

In November 2014, Dr. Peter Schielen from the Netherlands and I were searching for a photograph of Dr. J.M.G. Wilson, known as Max. Max was first author of the 1968 World Health Organisation monograph Principles and Practice of Screening for Disease [1]. Described as a public health classic and landmark publication [2], surely there was a photograph of one of the authors somewhere on the Internet? The answer appeared to be no. There was, however, an appreciation of Max in the Royal College of Physicians Munk’s Roll, Lives of Fellows (obituaries), Volume 12 [3]. This was written by Walter Holland, Professor of Epidemiology at St Thomas’ Hospital, London and by then at the London School of Economics, LSE. A few exploratory enquiries showed that Walter, now in his 80s, was still working one day a week at the LSE and was happy to be telephoned. He had known and worked for a short time with Max and was still in contact with his elderly widow, Mrs. Lallie Wilson, whose address Walter kindly shared with me. And so a letter typed in a large font for ease of reading was posted to Mrs. Wilson in Scotland wondering whether she could help with a photograph which might be suitable for the International Society for Neonatal Screening for use in a presentation or a publication or perhaps the Society’s website. Then, I waited. One evening, a telephone call came through. Stephen Wilson, the middle of Max and Lallie’s three sons was responding on behalf of his Mum. She was very willing to find a photograph of her husband. A talented artist, she also had a sketch of him. Both were kindly posted for scanning or photographing as required and then duly returned. And so, the first thread of Max’s life was picked up. The 10 principles contained within the Principles and Practice of Screening for Disease have reached almost biblical significance, likened to the 10 commandments (Figure 1). How did fate cause Max to come to co-author it with Dr. Gunnar Jungner from Sweden?

## 2. Early Life

**Figure 2:**
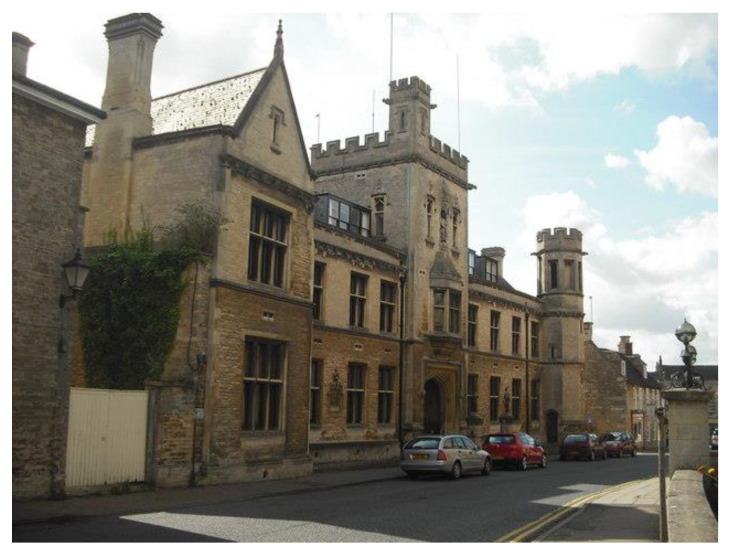
Oundle School.

Max was probably always destined to be a doctor or a lawyer. He was born on 31 August 1913 in Edinburgh, Scotland, to a father, James Thomas Wilson, who was a professor of anatomy and at that time on a sabbatical from Sydney, Australia. Max was the youngest of seven children, four girls and three boys. The family returned to Sydney and stayed in Australia until Max was seven years old. When his father was appointed as a professor of anatomy at Cambridge University, they returned to England. Max first attended Kings College Choir School in Cambridge and then Oundle School, Northamptonshire (Figure 2), following in the footsteps of his older brothers Douglas and John. Approximately 45 miles from Cambridge, it was then a boarding school for boys. The school had experienced expansion and investment under the visionary leadership of its famous headmaster, William Sanderson. His innovations put Oundle School at the forefront of science and engineering education, establishing a reputation for which it is still renowned today [4].

## 3. University

The three brothers—Douglas, John and Max—all attended St John’s College, Cambridge, where John studied law and Douglas and Max medicine. Douglas and Max were both taught anatomy by their father, a most meticulous man. In order not to show favouritism to his sons, he gave them both rather a hard time in class. Max graduated in 1938. The decision to study medicine was perhaps the first fork in the road or turning point in Max’s life that led to his writing his part of the Principles and Practice of Screening for Disease.

## 4. World War 2

Shortly after graduating in 1938, Max joined the Royal Army Medical Corps as a war substantive Captain and worked in a military hospital in Nigeria. He wanted to be part of the action and returned to England to join the 225th Para Field Ambulance. He was dropped in Normandy in the very early hours of D-Day, 6 June 1944. He tended many wounded soldiers in Main Dressing Stations before they were all withdrawn back to England in September 1944 [5] p395.

## 5. More Turning Points

Max met Lallie Methley at a social event towards the end of the second world war in Grantham, Lincolnshire. Lallie was working at the British Manufacture and Research Company (BMRC), where she quality checked cannon manufactured there for Spitfire planes to replace their now inadequate machine guns. By the end of the war, Max had developed a mild case of tuberculosis and went to Davos, Switzerland, to convalesce. Lallie had joined the post-war effort to rebuild Germany and, from there, visited Max in Switzerland and their future life together was sealed.

Max and Lallie had three sons—Andrew, Stephen and Philip—and by 1954, Max and his young family were living at number 49 Ann Street, Edinburgh, the city of his birth. Ann Street is amongst the most prestigious Georgian streets in Edinburgh’s New Town built in the 19th century. Max had a post at a small Edinburgh Hospital, where he had become a consultant physician. He gained a temporary lectureship at the Western General Hospital. He had hoped for further progression there, yet this was achieved by someone else. Dissatisfaction set in. Opposite the family home lived an anaesthetist, Dr. Harold Griffiths, with whom Max had become friends. The two men fished for salmon together and Mrs Griffiths had looked after the three little boys on occasion. Dr. Griffiths had been born in Rawalpindi and educated in Murree and Calcutta, all then located in India, and Max’s sons suspect this friendship may be have been relevant when their father looked for work elsewhere. Elsewhere turned out to be the Doom Dooma Tea Gardens in Assam, India, where Max became resident Principal Medical Practitioner for the tea plantation workers.

**Figure 3:**
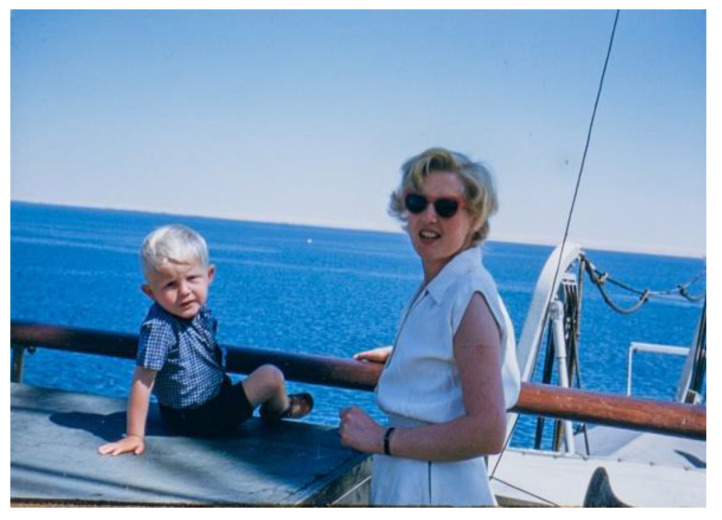
Stephen Wilson and Mrs Wilson aboard the Polish ship Batory en route to India.

Thus, in October 1954, the family set sail for Assam, India, on the Polish ship, the Batory, known as the ‘lucky ship’ (Figure 3), having avoided destruction from torpedoes during the war. 

This move could be considered a further turning point in Max’s life. Before the tea plantations, the Doom Dooma estate had once been covered in jungle and mainly populated by elephants. Although its true origin is shrouded in mystery, that curious name Doom Dooma was thought by some to represent the sound of the elephants’ footfall when they ran through the jungle. The Assam tea gardens lie in the lowlands on either side of the Brahmaputra River, south of the eastern Himalayas, and are amongst the largest tea plantations in the world. Of these tea gardens, Doom Dooma is considered to be the most beautiful across the globe.

**Figure 4:**
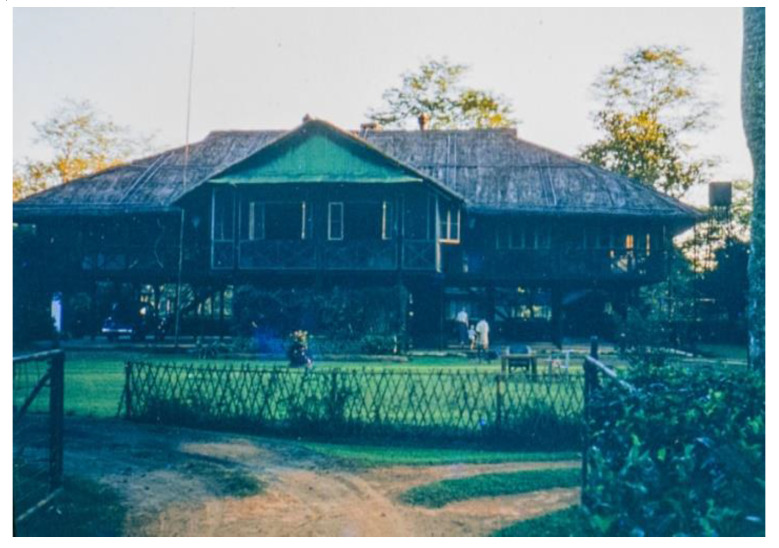
Principal Medical Officer’s House, near the Dibru River, where the Wilson family lived.

The family lived in a compound on the edge of Doom Dooma town in a bungalow on stilts (Figure 4), typical of the area, allowing the breeze to blow beneath keeping them cool and to protect them from flooding and wild animals.

**Figure 5:**
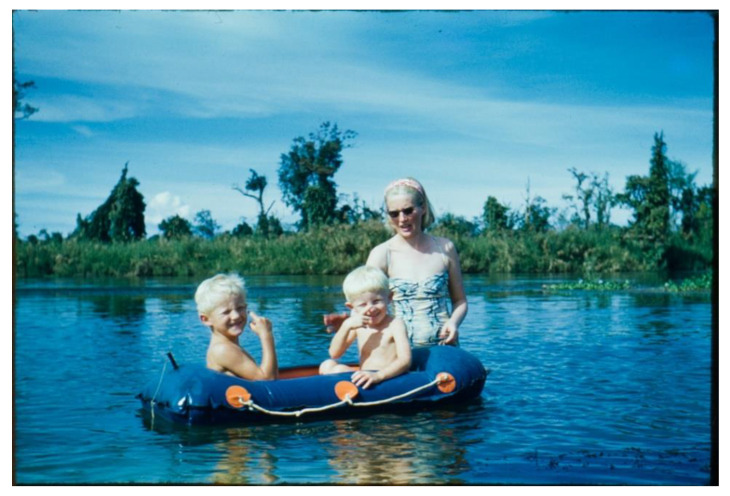
Mrs. Wilson and sons Philip, left, and Stephen, right, enjoying the Dibru River.

Max worked at the Beesakopie Central Hospital approximately a mile away and also visited smaller clinics. He was driven around the plantations in a Land Rover by Abdul. The house was close to a tributary of the Brahmaputra River—the Dibru River, where the family went for picnics and swam unaware of the crocodiles lurking nearby (Figure 5). Naga tribes people sometimes wandered through the tea plantations selling brightly coloured textiles and crafts and Lallie purchased several examples. Max’s eldest son Andrew went to boarding school in Darjeeling, whilst the younger sons Philip and Stephen stayed at home with Lallie. 

## 6. A Swedish Connection

**Figure 6:**
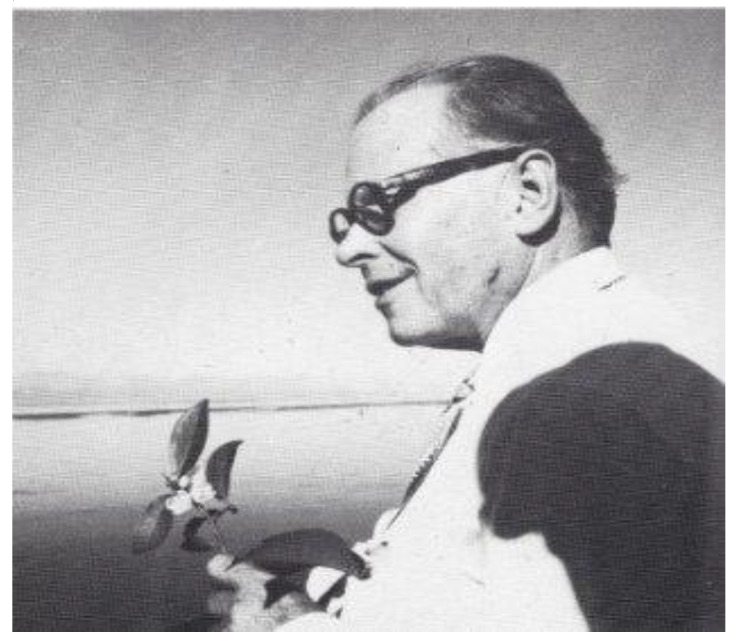
Professor Höjer looking out over the Brahmaputra River with a sprig of camellia sinensis (tea).

Dr. Axel Höjer and his wife Signe Höjer were key figures in the Swedish welfare state. Axel was Director of the Swedish Medical Board between 1935 and 1952. In a 1948 report, he argued that Swedish public health care should now shift its focus to disease prevention, monitoring the healthy rather than managing the sick—in other words, screening. Significantly, Höjer’s report recommended setting up a pilot population health screening project. This did not take place before Axel retired. In 1956, he accepted a position of Professor of Preventive Social Medicine in Assam Medical College, Dribrugarh (Figure 6).

**Figure 7:**
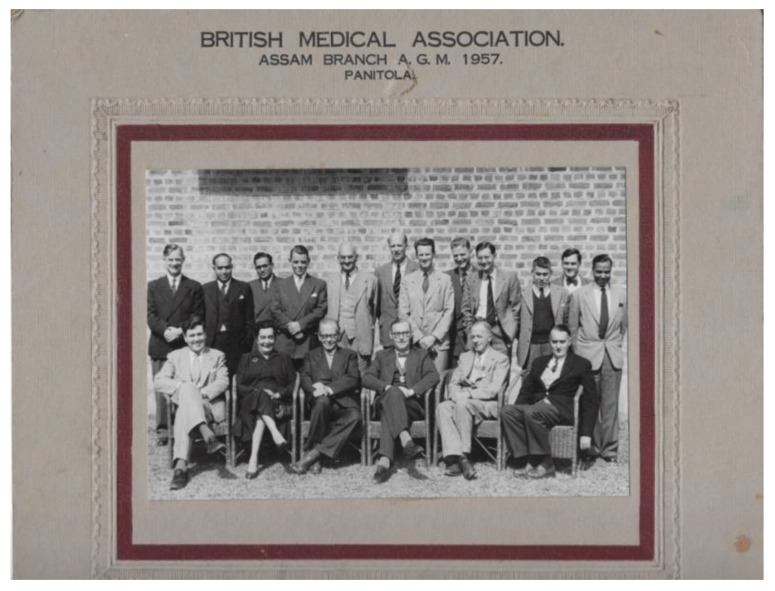
Dr. J.M.G. Wilson, 7th from the left back row and Prof Höjer, 3rd from left front row, at a meeting of the Assam branch of the British Medical Association in Panitola, Dribrugarh.

**Figure 8:**
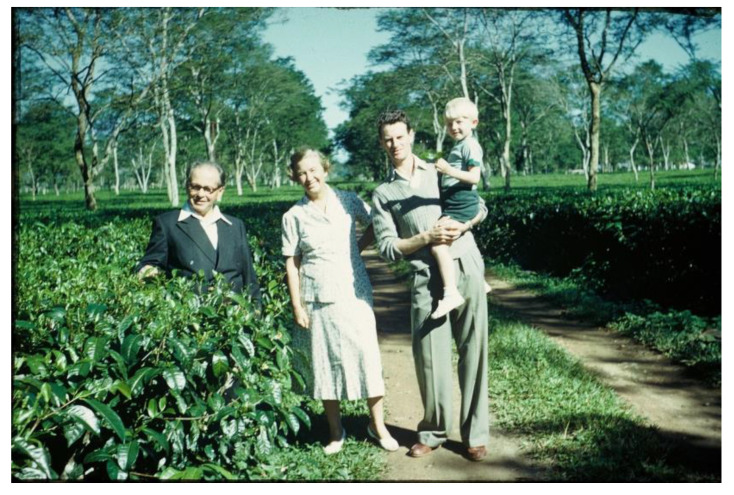
Axel and Signe Höjer with Dr. J.M.G. Wilson and his son Philip in the tea garden.

**Figure 9:**
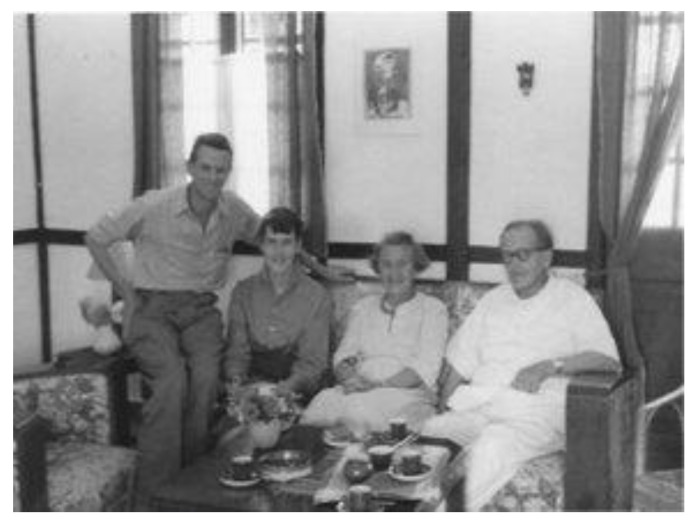
Dr. J.M.G. Wilson with his niece Mary and Signe and Axel Höjer taking tea together in the Principal Medical Officer’s house.

He moved with his wife Signe into a small house surrounded by tea plantations. Max met Axel at meetings of the Assam branch of the British Medical Association in Panitola, Dribrugarh (Figure 7). The two families became friends spending time out and drinking tea together (Figure 8 and Figure 9).

However, clouds were forming on the horizon. After three years, Lallie had become unsettled with her life in India and wanted to return to the UK. The heat and humidity in Assam were unbearable, the ‘English’ food made by the cooks unappetising and the expatriate life of mahjong, gin drinking sessions and servants unappealing. She endeavoured to solve the food problem by visiting local markets with their colourful array of fruits, vegetables and spices, setting up a temporary kitchen in the bungalow with paraffin stoves and a pressure cooker and cooking the family meals herself. This was not what a memsahib should do. Lallie would say that Dooma Dooma was aptly named—it echoed the English word doom and how she felt. Max was opposed to the management’s policy of sacking sick and injured workers and replacing them with cheap new labour. After an earthquake had destroyed many of the native workers’ flimsy houses and maimed or killed a number of them, there was a heated and confrontational meeting with management. The native workers felt abandoned and neglected and Max had wished to be present at the meeting in case a doctor was needed. The management advised against it and Lallie did not want him to attend.

**Figure 10:**
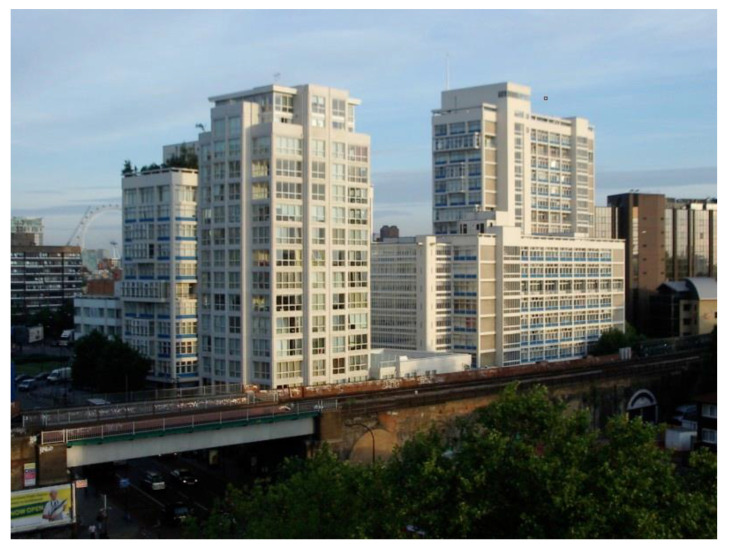
Ministry of Health at Alexander Fleming House in London, 1960–1989. The building was designed by Hungarian architect Erno Goldfinger.

Lallie decided to bring the children home to Scotland when the school term at Andrew’s boarding school had finished. In his discussions with Axel Höjer, Axel had recommended to Max a career in public health. This perhaps represented another significant turning point, another fork in the road. Subsequently, Max secured a post in public health at the Ministry of Health in London as Senior Medical Officer based at Alexander Fleming House (Figure 10). 

Lallie came down south from Scotland with her sons to be with Max—initially at Cerne Abbas in Dorset, and then at his brother John’s house in Mickelham, Surrey—whilst they looked for somewhere to live. The first house was in Buckland and the next at Redhill—only 30 minutes away by train from work. His boss was Sir George Godber, Deputy Chief Medical Officer, who ‘had a habit of selecting young people, helping them in their career, and using their talents.’ [6] 

In March 1961, George, now the Chief Medical Officer at the Ministry of Health, was appointed as the UK representative on the Executive Board of the World Health Organisation (WHO). George had become aware of the growth of population screening in the USA and Canada—cervical screening in particular. Encouraged by George, Max went on a WHO travelling fellowship in the following year, 1962, to the USA and Canada in order to study multiphasic population screening. Multiphasic screening or multiple screening are terms describing the application of several tests or examinations at the same time for different disorders. These terms could be used to describe newborn screening today. Prior to the transatlantic trip, Max sought advice and information from Dr. Walter Holland (who was to prove to be so important in the genesis of this article) as to who to visit on his trip to North America.

## 7. Why North America?

The UK National Health Service, implemented in 1948, provided health care free at the point of delivery for all according to need and not wealth. The great question for the Ministry of Health (i.e., the National Health Service) was how far to accept the claims being made for health screening as had been developed in North America [7] p202. In April and May 1962, Max visited 97 people in 15 cities in the East, Mid-West and West USA and also Vancouver, Canada. Whilst too many to acknowledge in this paper, these people included:

Dr. Lester Breslow, Division of Preventive Medical Services, California State Health Dept.;
Dr. Morris Collen from Kaiser Permanente Medical group, Oakland, California;
Dr. Quentin Remein, Washington, D.C.;
Dr. Morton Levin, Kress Institute, Roswell Park Hospital, Buffalo, NY.

## 8. Findings from the USA

Back in 1962, mass screening had been carried out in the USA for nearly 15 years. However, it had not proved possible to make a detailed assessment of its value as a public health intervention. In Max’s opinion, the reasons for this problem were several fold:

(a) The mobility of the population studied;
(b) The difficulty of aligning a public health screening programme, in which individuals would be identified, with the private medical sector in whose care the follow up typically took place;
(c) The difficulty in obtaining a sufficiently representative population to take part in the scheme.

Max reported his findings and conclusions in the Lancet [8]. He considered that without a sound knowledge of the natural history of the conditions being screened, trouble could soon arise. Additionally, and perhaps more controversially, his view was that unless treatment reduced illness, only harm would be done by bringing the condition to the patient’s attention. In a 1994 paper written in retirement [9], Max would refer once again to some of the issues of the screening surveys which took place in the USA in the 1950s. He noted that lack of validation of individual screening tests, lack of knowledge of the outcome of the disorder following early detection and lack of arrangements for medical follow up resulted in an inability to evaluate any benefits of health screening. Preventing ill health was a lifelong concern of his.

## 9. Timelines and Significant Meetings

Outside the USA, health screening was possibly first discussed on the international stage at the 1957 Symposium on ‘Public Health Aspects of Chronic Disease’ Amsterdam, the Netherlands, sponsored by the Regional Office for Europe of the WHO in collaboration with the government of the Netherlands. George Godber was present at the meeting as the Member nation representative for Great Britain. The USA pioneer in screening, Dr. Lester Breslow, was an invited lecturer for the meeting. Whilst he was unable to attend, his paper ‘Early detection of asymptomatic disease’ was introduced by an attendee from the Netherlands on his behalf. Most of his five conclusions in the paper, which Godber will have heard, still apply today and include ‘Multiphasic screening is a combination of several disease detection tests … applied to large groups of apparently well persons. The test results if positive, suggest specific need for diagnosis; they do not constitute diagnosis,’ ‘Multiphasic screening contributes to good medical practice …,’ Multiphasic screening provides an excellent opportunity for health education’ and ‘Multiphasic screening offers public health departments a realistic approach to the prevention of chronic illness.’

Some years later, a WHO Regional Committee for Europe, 14th session, was held in Prague, Czechoslovakia in 1964. The technical discussion for this meeting was entitled ‘The pre-symptomatic diagnosis of disease by organised screening procedures.’ The man Max was now working for, Sir George Godber, was Chair of the session and Dr. Gunnar Jungner an advisor representing Sweden. He was Associate Professor and Head of the Department of Clinical Chemistry at Sahlgrenska Hospital at the University of Gothenburg. Gunnar introduced a paper discussing automated chemical screening and the Värmland Health Screening Project.

**Figure 11:**
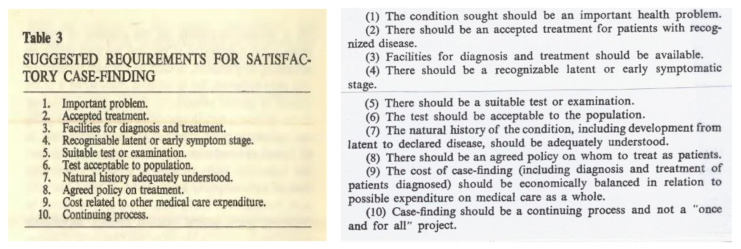
Left, requirements for screening presented by Max to the Oxford meeting in 1965. Right, almost identical *Principles of Early Disease Detection* shown in the 1968 World Health Organisation (WHO) monograph No 34 [1].

The following year, on 7 July 1965, Max met Gunnar Jungner [10], possibly for the first time, at Magdalen College, Oxford, at a conference, ‘Surveillance and Early Diagnosis in General Practice’. Gunnar spoke on chemical health screening and also referred to the Värmland project in Sweden. Max spoke to the title ‘Some principles of early diagnosis and detection.’ It is clear that Max had by now formulated his conclusions on the principles of population screening and his list of 10 criteria that were presented are virtually identical to those in the paper and monograph that were later written for the WHO (Figure 11).

It took another meeting for the way finally to be paved to writing the monograph. Max gave a report to the WHO Conference ‘Early Detection of Cancer’, Oslo, Norway, 15–19 November 1965. Significantly, the meeting was attended by Dr. Fred Grundy, Assistant Director General, WHO. It was Dr. Grundy who subsequently invited Max and Gunnar to write a briefing paper on the early detection of disease. As the authors wrote [11] ‘… we found we were unable, with a subject matter that covers not only a great deal of medicine but is also breaking new ground rapidly, to write a short paper and it has now grown to its present length’. The paper was received into the WHO library, Geneva, on 4 May 1967 and published the following year [1] as number 34 of the WHO’s Public Health Papers.

## 10. The Men

**Figure 12:**
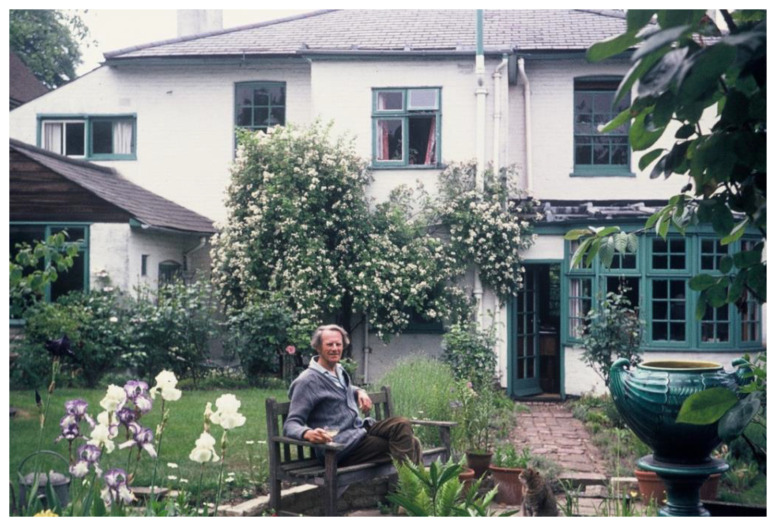
Max relaxing in the garden at Redhill. Gunnar Jungner visited him here in the 1960s.

During the writing of the Principles and Practice paper, Max and Gunnar visited each other in Sweden and England. An enduring memory of Max’s sons is Gunnar’s surprise at seeing Lallie Wilson mowing the lawn when on a visit in the mid-1960s to the Wilson’s family home in Redhill, Surrey (Figure 12). In his collection, Max had a photograph of Gunnar standing with Axel and Signe Höjer somewhere in Sweden. It is not known whether all four met together in Sweden.

## 11. The Man from the Ministry

In his role as Senior Medical Officer at the Ministry of Health, Max became responsible for commissioning health research. He was also invited to give reports and briefing papers at the WHO and other meetings where population health screening was discussed:

1. 1968, London—Meeting to discuss the dietary treatment of PKU;
2. 1969, London—Conference on the study of inborn errors of amino acid and carbohydrate metabolism in infants;
3. 1971, Geneva—WHO Technical Discussion ‘Mass Health Examinations: a review of the subject’ compiled and written by Max;
4. 1975, Erskine, Scotland—Conference of Chief Administrative Medical Officers, scientific session on screening.

In 1976, Max was offered the post of Deputy Chief Medical Officer in London (Sir George Godber had retired as CMO in 1973). By this time, he had become disillusioned and declined the invitation. Instead, he took up a research fellowship post with his old friend Dr. Michael Heasman at the Scottish Health Service Common Services Agency in Edinburgh. Rather than living in Edinburgh again, he chose to live in Musselburgh on the East coast of Scotland. He retired in 1978 aged 65 and died nearly 30 years later in a nursing home in Musselburgh on 31 December 2006 aged 93. After his death, his wife Lallie continued to live in the family home in Musselburgh.

## 12. Recollections

**Figure 13:**
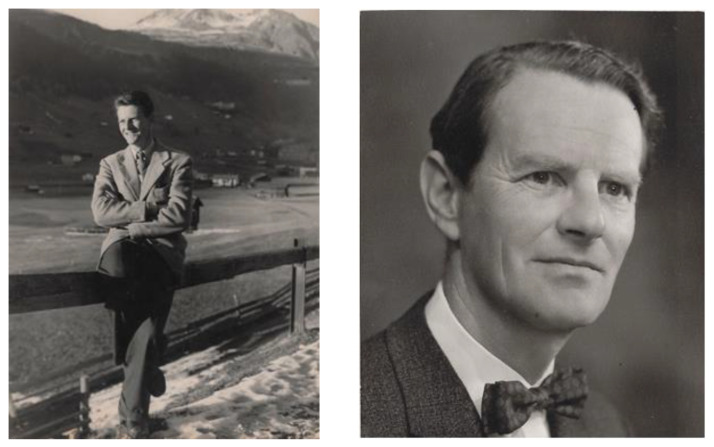
Max Wilson in Davos, Switzerland, circa 1946, left, and in the 1960s, right.

Max’s sons remember him (Figure 13) as a quiet, studious man who loved books, music, history and outdoor activities. He enjoyed reading stories to them in front of the fire in their cottage in the Scottish Highlands. He was exceptionally hard working, seeking perfection in all he did. During the time they lived at Redhill, London, and Max worked at the Ministry of Health, he left for work on the train to central London at approximately 07:00 in the morning, returned at approximately 19:00, had his evening meal and worked for another few hours. In sunny weather, he would set up an old wallpapering table in the garden, spread out all his papers and work outside.

Both Max and Gunnar’s children had not been aware until recently of the significance of their fathers’ work and especially that the names of Wilson and Jungner are so often quoted in conversations about population screening to this very day. Their legacy lives on. Walter Holland wrote [3] of Max ‘His true contributions were often not sufficiently appreciated due to his retiring nature’.
